# Diversity in eMental Health Practice: An Exploratory Qualitative Study of Aboriginal and Torres Strait Islander Service Providers

**DOI:** 10.2196/mental.7878

**Published:** 2017-05-29

**Authors:** Jennifer Bird, Darlene Rotumah, James Bennett-Levy, Judy Singer

**Affiliations:** ^1^ University Centre for Rural Health—North Coast School of Rural Health University of Sydney Lismore Australia

**Keywords:** eMental Health, Aboriginal and Torres Strait Islanders, social and emotional wellbeing, health education, health promotion, mental health, indigenous health services, culturally appropriate technology, internet, implementation, training

## Abstract

**Background:**

In Australia, mental health services are undergoing major systemic reform with eMental Health (eMH) embedded in proposed service models for all but those with severe mental illness. Aboriginal and Torres Strait Islander service providers have been targeted as a national priority for training and implementation of eMH into service delivery. Implementation studies on technology uptake in health workforces identify complex and interconnected variables that influence how individual practitioners integrate new technologies into their practice. To date there are only two implementation studies that focus on eMH and Aboriginal and Torres Strait Islander service providers. They suggest that the implementation of eMH in the context of Aboriginal and Torres Strait Islander populations may be different from the implementation of eMH with allied health professionals and mainstream health services.

**Objective:**

The objective of this study is to investigate how Aboriginal and Torres Strait Islander service providers in one regional area of Australia used eMH resources in their practice following an eMH training program and to determine what types of eMH resources they used.

**Methods:**

Individual semistructured qualitative interviews were conducted with a purposive sample of 16 Aboriginal and Torres Strait Islander service providers. Interviews were co-conducted by one indigenous and one non-indigenous interviewer. A sample of transcripts were coded and thematically analyzed by each interviewer and then peer reviewed. Consensus codes were then applied to all transcripts and themes identified.

**Results:**

It was found that 9 of the 16 service providers were implementing eMH resources into their routine practice. The findings demonstrate that participants used eMH resources for supporting social inclusion, informing and educating, assessment, case planning and management, referral, responding to crises, and self and family care. They chose a variety of types of eMH resources to use with their clients, both culturally specific and mainstream. While they referred clients to online treatment programs, they used only eMH resources designed for mobile devices in their face-to-face contact with clients.

**Conclusions:**

This paper provides Aboriginal and Torres Strait islander service providers and the eMH field with findings that may inform and guide the implementation of eMH resources. It may help policy developers locate this workforce within broader service provision planning for eMH. The findings could, with adaptation, have wider application to other workforces who work with Aboriginal and Torres Strait Islander clients. The findings highlight the importance of identifying and addressing the particular needs of minority groups for eMH services and resources.

## Introduction

### Background

Addressing the mental health and wellbeing needs of Australian Aboriginal and Torres Strait Islander people is a priority of the Australian Federal Government [1,2]. The high levels of psychological distress, mental health disorders and related conditions, alcohol and other drug misuse, and suicide among indigenous populations in developed countries like Australia, New Zealand, Canada, and the United States are well documented [3-6]. Mental health profiles for indigenous populations living in postcolonial societies are largely attributed to the complex and cumulative impacts of the colonization process on the lived experience of indigenous people [1,7-9].

Yet indigenous peoples around the world, in both developing and developed countries, are among the least likely to have access to mental health and related services [10]. Providing accessible and effective mental health and wellbeing services to minority indigenous populations in developed countries is challenged by a variety of factors:

- Geographic distance from services for indigenous people living in remote locations [10]
- Lack of coordination between health and other government sectors that mitigates against addressing broad social, cultural, and historical determinants of health and wellbeing [7,11]
- Discord between biomedical and indigenous understandings of mental health, social and emotional wellbeing, and the treatment and management of mental ill health [10,7,11]
- Lack of cultural congruence in service delivery and lack of cultural competence of mental health staff [10,11]

In Australia the term *social and emotional wellbeing* offers an alternative and more positive term to mental health. The term social and emotional wellbeing emerged in Australia in the 1980s as a reaction against and an alternative to conventional psychiatric mental health terminology [7]. The term attempts to express a more holistic conception of health and mental health and includes the social, political, and historical determinants of health.

The Australian Federal Government’s eMental Health Strategy [2] aims to increase availability and accessibility to low- to medium-intensity mental health services for all Australians through the promotion of eMental Health (eMH) resources. Examples of eMH resources that have garnered support for Aboriginal and Torres Strait Islander people are the *AIMhi Stay Strong* app for mobile tablets [12], which is a culturally specific practitioner-led therapeutic tool; the *Mindspot Clinic Indigenous Wellbeing Course* [13], which is a culturally adapted online assessment and treatment program; and *beyondblue* [14], which is a prominent website for depression and anxiety that includes a campaign on the impact of racism on mental health.

Promoting eMH resources and training service providers working with Aboriginal and Torres Strait Islander people and Aboriginal and Torres Strait Islander service providers in the use of eMH resources is one priority within the strategy.

### Services and Workforces

A model of wellbeing by Milroy [15] describes the range of services that impact on the social and emotional wellbeing of Aboriginal and Torres Islander people as including:

- Inadequate training for the level of responsibility expected
- prevention, promotion, and early intervention services
- comprehensive primary health care
- secondary, tertiary, and specialized health care
- government sector (housing, employment, education, justice, recreation, disability, welfare, and social services)

Many of these services, particularly in the government sector and some sections of the health sector, are not formally recognized or funded as delivering mental health services, yet they support the social and emotional wellbeing of Australian Aboriginal and Torres Islander people [7].

The Aboriginal and Torres Strait Islander service provider workforce is a small community-based cross-sectoral workforce that plays an essential role in supporting the social and emotional wellbeing of Aboriginal and Torres Strait Islander people. They work mainly in either the health or community services sector. In Australia, Aboriginal and Torres Strait Islander health workers carry out a variety of roles including cultural brokerage between clients and the health system, health promotion, environmental health, clinical, and community work [16]. Similar workforces in other developed countries are Maori community health workers in New Zealand [17] and community health representatives who work with American Indians and Alaskan Natives in Canada and the United States [18]. Community workers are employed across a range of community-based social welfare services with the overall aim of improving social inclusion and social functioning for their clients [19].

As minority workforces working with a minority population, Aboriginal service providers face particular challenges:

Inadequate training for the level of responsibility expected

- Excessive client workloads
- Clients with extremely complex problems; volatile clients; violence; mental health; drug and alcohol; and grief, loss, and trauma
- Blurred boundaries between work, community, and private life
- Racism [20,21]

### eMental Health Implementation Studies

Despite the potential that eMH offers to improve access to mental health interventions, the implementation of eMH by health services and health professionals has been reported as uniformly low, both in Australia and Europe [22,23]. Research efforts in the eMH field to date have primarily focused on establishing an evidence base for the effectiveness of individual eMH resources, particularly low-intensity online symptom-focused treatment interventions. Less focus has been placed on investigating how to facilitate the implementation of eMH by health services or how to improve the uptake of eMH by users and help seekers [24]. Such is the gap between effectiveness and implementation that in 2015 a large-scale, 5-year empirical implementation study was launched across 15 European regions to investigate the factors that hinder or promote the use of Internet interventions in practice [23].

Implementation studies of eMH in health and related workforces are generally more theoretical than empirical [25]. Theoretical frameworks and models typically focus on describing enablers and barriers to the implementation of technological innovations within workforces [26,27]. How individual practitioners might integrate eMH resources into routine practice is one factor identified in the eMH implementation literature [24]. Puszka et al [25] describe the degree to which eMH resources can be integrated into usual practice as one important factor in mediating use. Yet within the eMH implementation literature there are, at time of writing, no studies that empirically report on the manner in which health professionals are using eMH resources in their routine practice.

This study aims to extend the findings of two previous studies into eMH implementation within the particular context of service delivery to Aboriginal and Torres Strait Islander people [25,28]. These previous studies investigated different stakeholder perspectives on the potential implementation of eMH resources among Aboriginal and Torres Strait Islander health and community services workers. One investigated the perspectives of managers of health services on the requirements for implementing eMH resources [25]. The other investigated trainer/consultant observations about the uptake of eMH resources by Aboriginal health professionals during participation in an eMH training and consultation program [28]. Both studies found sets of interconnected enablers and barriers to the implementation of eMH resources that included attitudes and skills of the individual worker, the organizational systems within which they work, their clients, and the design of the eMH resources themselves.

There are no studies to date that have investigated the manner in which Aboriginal and Torres Strait Islander health and community services workers have integrated eMH resources in their routine practice, as reported by practitioners themselves.

### Purpose of the Study

The purpose of this exploratory study is to investigate how Aboriginal and Torres Strait Islander service providers in one regional area of Australia, northern New South Wales, have used eMH resources in their practice after participating in an eMH training program and what types of eMH resources they used.

## Methods

### Setting

The project within which this study took place has been engaged in the promotion and training of eMH with Aboriginal and Torres Strait Islander service providers for the last 3 1/2 years in a regional/rural area of Australia, northern New South Wales. The project is one partner in the Australian Federal Government’s national e-Mental Health in Practice project.

A previous paper from this project examined observations of the uptake of eMH by Aboriginal and Torres Strait Islander service providers during training from the perspective of the trainers/consultants [28]. In this study, a sample from the same population was interviewed in their workplaces 4 to 8 months after training to investigate the use of eMH in participants’ routine practice.

Ethics approval was gained from the North Coast New South Wales Human Research Ethics Committee (076) and the Aboriginal Health and Medical Research Council Human Research Ethics Committee (955/13).

### Recruitment

The population from which the study sample was drawn was 28 Aboriginal and Torres Strait Islander service providers who had participated in an eMH training program that involved either a 2- or 3-day eMH training workshop followed by up to 6 monthly skills-based follow-up consultation sessions [28].

Purposive sampling was used to recruit participants from this population to ensure representation across different ages and genders, a range of services and locations, and varying degrees of engagement with the training program.

### Sample

The 16 Aboriginal and Torres Strait Islander service providers interviewed in this study were employed in either the government health sector (n=4), mainstream nongovernment community service organizations (n=6), or Aboriginal and Torres Strait Islander–controlled health and community service organizations (n=6). Most workers (15/16) interviewed were employed in identified Aboriginal and Torres Strait Islander positions, and half (8/16) were female. Job titles demonstrate employment in a broad cross-section of positions that included health education, wellbeing, mental health, family services, disability, and alcohol and other drugs. Participants in this study were not trained mental health professionals.

All but one interviewee had completed or was currently enrolled in a formal qualification relevant to their position. Qualifications ranged from vocational certificates or diplomas (n=26) to postgraduate university master’s degree (n=1). Some interviewees held multiple qualifications at varying levels. The most frequently reported fields of study were Aboriginal health (n=5), community services (n=5), counseling (n=4), and alcohol and other drugs (n=4). One participant was enrolled in a psychology degree program at time of interview. In addition to formal courses, interviewees reported completing a range of short training courses relevant to eMH, the most popular being *Mental Health First Aid* [29] and suicide prevention.

### Data Collection

Two interviewers, an external qualitative researcher (JB) and an Aboriginal health professional/doctoral student (DR), conducted 16 semistructured qualitative interviews of approximately 60 minutes duration. The interviews were conducted 4 to 8 months after the completion of an extended eMH training program that included workshops and skills-based follow-up consultation sessions. At the commencement of each interview, participants were asked to describe the service within which they worked, their job title, their level of qualification, their relevant history of short course training and professional development, and their role. The interview questions focused on 3 main areas of investigation: participant experiences of the training and consultation sessions, participant uptake of eMH resources subsequent to the training, and participant needs for and access to supervision.

### Data Analysis

Thematic analysis of the qualitative data relevant to this study from the 16 interviews was conducted using the method described by Braun and Clarke [30]. They describe a 6-step process of thematic analysis: familiarize with the data, generate initial codes, search for themes, review themes, define and name themes, and produce a report.

Two researchers (JB and DR) separately read a random sample of 8 transcripts and identified relevant data extracts from each interview. The data extracts were then peer reviewed and amended. Each researcher separately created a set of initial codes for the 8 sample interviews. The codes were peer reviewed and amended. The initial codes were then applied to the remaining 8 transcripts and tested, discussed, and amended by the two researchers. All the data extracts were then collated under the final codes. Each theme was analyzed and coded for subthemes.

## Results

### Context of Practice

Participants described their roles as having a broad scope that reflected the social determinants of social and emotional wellbeing of their clients. Participants described working with clients in the areas of housing support, drug and alcohol, domestic violence, criminal justice diversionary programs, child protection, and family support. They engaged in health education, crisis management, counseling, and social support and inclusion. One participant referred to himself as a *jack-of-all-trades* given the broad scope of problems and issues he encountered with his clients.

Participants highlighted the number of clients who present to them in crisis and with complex needs and the impact on their client load of referrals from mainstream agencies. They spoke of the unique and complex relationships they have with the communities to which they belong and with whom they work.

Participants all worked in community-based organizations and with only one exception engaged with their clients either in their homes, in community settings away from their place of employment, or in outdoor areas connected to their workplaces. One participant worked in a medical community outreach program. Participants engaged with clients either individually for outreach support or counseling; in families for family outreach support; or in groups for rehabilitation, education, and support.

### Purpose of eMental Health Practice

**Figure 1:**
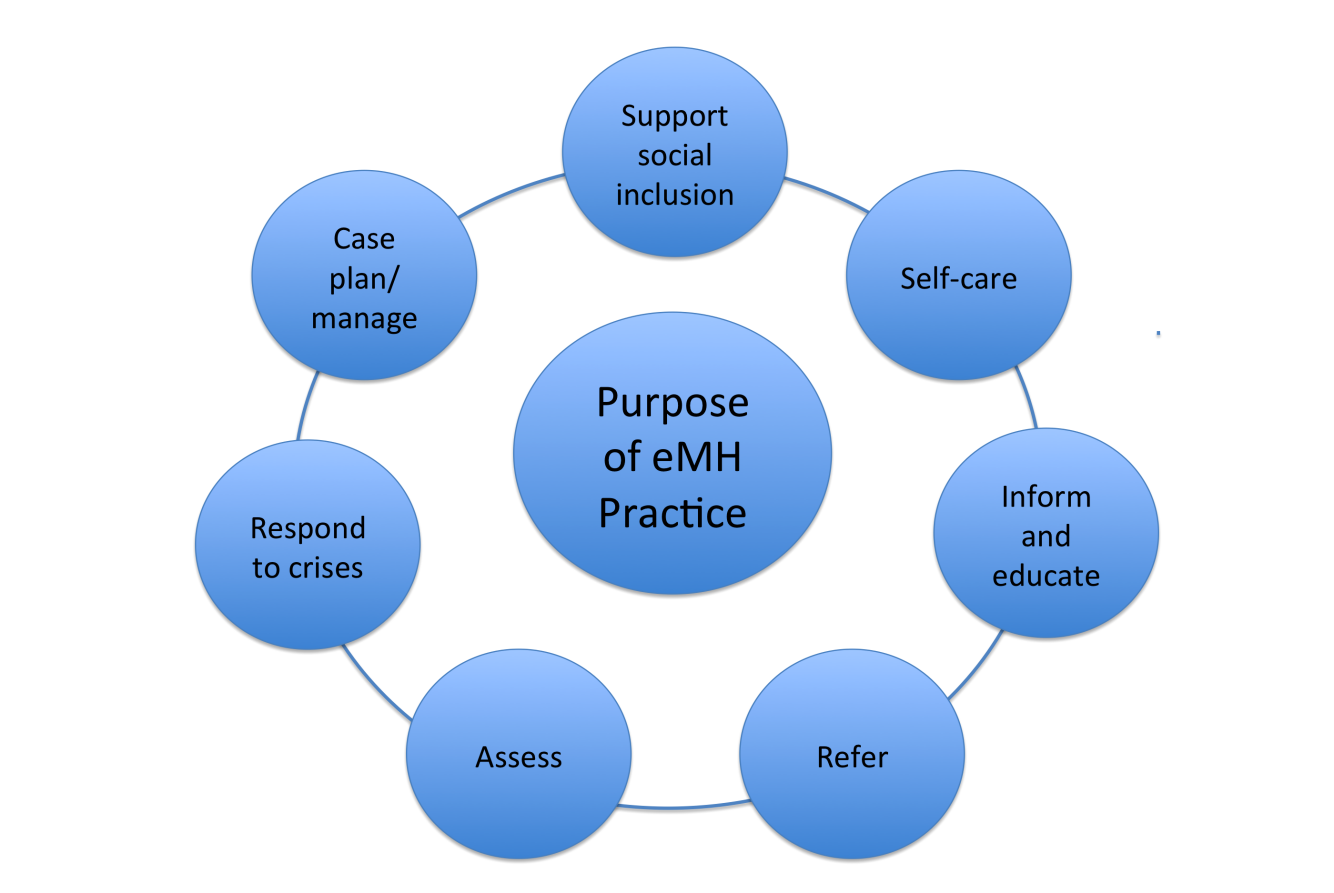
Purpose of eMental Health practice.

Of the 16 participants interviewed, 9 had implemented eMH resources into their practice with their clients since completing an eMH training program. Figure 1 describes the variety of uses to which they put eMH resources with their clients.

#### Inform and Educate

There were various ways that workers used eMH resources to provide information and education to their clients. Workers engaged in information sharing and education with individual clients and with groups of clients. These events were scheduled or spontaneous, one-off or ongoing. Workers used eMH resources with their clients or referred their clients to relevant information or education resources.

We used the Ice one [YouTube clip] in a good session because it tells you about how when ice [crystal methamphetamine] is injected or smoked and it goes into your brain what it does to your brain and what it does to your body.

There is a quit smoking one. Like a game show about quitting smoking and it's indigenous-specific so we use that with our clients.

...if you're talking to a client, you can speak to him and you can say, “Okay, well, look up this program,” and maybe we'll get some information, we'll talk about it.

In addition to informing and educating clients, one worker described the role that Facebook plays in informing and educating other workers and the community about resources and programs that are available.

A lot of that information is actually getting out there via Facebook. I think beyondblue has got a great program. Mindspot comes up a lot. There's programs for people with cannabis misuse. There's all sorts of stuff you see coming and I'll always share that stuff, because it might be someone that's sitting there and thinking “I'm not comfortable going to my Aunty up the road.” That gives them a choice.

#### Assess

Depending on the type of service in which they work, workers undertook a variety of assessments with their clients. Assessments could range from mandatory mental health assessments required by child protection authorities to intake assessments tailored to the particular service, for example, drug and alcohol services or community-based wellbeing services. Assessments could be of individual clients or families, adults, or children. Assessments could be formal and worker-led or informal client-led self-assessments.

The workers who participated in this study had previously been trained in the use of a culturally adapted mobile app called *AIMhi Stay Strong*, which some participants were using for assessment.

We used to just sit at the desk...and I'm just asking them questions off the computer...I found [Stay Strong] a lot more engaging than sitting at the desk. We'd sit out in the open and do it, and they felt really comfortable.

What I find with the young fellas is that I don't have to do anything. I just turn it on and say, look...I explain to them what it is and what to do, because next time we meet, we can have a look at it again. They just do it themselves. Really simple...Then I'll check it and it's all done properly.

#### Case Plan or Manage

The majority of workers in this study worked in services that used case planning with clients. Workers managed an allocated caseload of clients. Workers used a variety of terms to describe the manner in which they managed their clients over time. These included service planning, goal setting, life coaching and checking in. Depending on the service, case planning may be a formal process mandated by the service or an informal ongoing check-in process.

Some participants reported using *Stay Strong* with clients as a case management tool. Particular mention was made of the goal setting function within this app.

We make a plan of what they want to achieve in the next 3 or 6 months and then we support them in that plan just as kind of coaches, life coaches, so that's my kind of role.

When I go down to see these clients, I just usually take the app with me and open it up. We look at it together and I say, “What's changed?”

We found out the younger ones like setting themselves up with goals. They were really looking forward to see what they can change in their life. They're like, ”Yeah, well, we're going to check this out next time we sit down,“ and go over it and see if they reached a goal or they're moving towards where they want to be.

#### Respond to Crises

Despite the majority of workers in this study having a case load and working within a case planning framework, many described their working lives as being driven by client crises, external demands from other services, and cultural or community responsibilities.

Going online, finding all these different [telephone] numbers...because there are times I wouldn't happen to make it back out [to a community], or there might be a drama, or dilemma, or something happened in the community. There are emergency numbers that you can access.

#### Refer to eMental Health Resources

Participants referred to a range of health and social services. They describe using technology to access the websites and contact details of agencies to which they can then refer their clients, refer clients to eMH resources like online programs and mobile apps, and refer clients to emergency telephone numbers and hotlines. Throughout the comments about this particular activity ran a theme of encouraging agency and independence in their clients.

Instead of me specifically using a tool for anxiety...I might go to beyondblue anxiety. I think if they actually want to do anything about it I wouldn't really have that conversation with them. I'd probably need to take them to a doctor.

Just mention it. If they [clients] choose to put it on there [onto their mobile phones] themselves, then they'll come back and go, ”I got one. It seems to be working good.“ ...then they'll show me what they've got or tell me how it's going, if they enjoyed it, or if it worked, or just tell me how it worked for them.

If my client tomorrow, if he's got a smartphone or if he's got an iPad, I would say, you should have a look at this by yourself or check it out and he can do all the stuff on it himself.

I sometimes will Google [with] clients, find information and numbers and things.

Part of my work, if I'm out in the community I can just say to the younger ones, or family members, ”There are websites that you can look at, also. It helps me to look at some numbers, like contact numbers, maybe, grief, or loss, or suicide, or whatever.”

#### Support Social Inclusion

Workers used eMH resources to engage with clients for the purpose of connecting them to relevant social and community services, building social skills, and supporting clients practically toward employment, education, and training.

I work with people who have mental illnesses. They might need help just with day to day stuff and also social stuff, so getting them connected into the community so that they have more support networks. I use different technology to achieve that. We all have an iPad, so we take that with us everyday and have lots of apps and access to Internet, videos, all sorts of stuff, music, just to help our participants get used to accessing the Internet services for information.

#### Care for Self and Family

Participants reported using eMH resources for themselves and their families to help manage stress and anxiety.

I think I actually applied to do a Mindspot one. I do experience anxiety sometimes. I guess it's more people need to be aware that they matter. It's that self-care stuff.

I used them for myself. The meditation one.

I can even use them with my own family members. When my brother-in-law's not well, I do that Stay Strong. I say to him, “Just have a look.”

The meditation one. I asked one of my aunties if she'd be interested, but she doesn't know how to use a computer, so I showed her on mine. She loved it.

### Types of eMental Health Resources

Participants reported using a variety of types of eMH resources with their clients. They used both culturally specific and mainstream resources, described below

#### Information Websites and Online Telephone Crisis and Support Services

Participants referred clients to popular mainstream mental health websites like *beyondblue* and *Mindspot* and referred clients to major mainstream online telephone crisis support services like *Lifeline.* The range of information websites and support services websites extended beyond the field of mental health and into health and welfare services.

#### Online Symptom-Based Treatment Programs

While participants were aware of online symptom-based treatment programs and some had promoted them to their clients, no one had embedded them into their practice. One participant had accessed an online treatment program for anxiety for their personal use but then disengaged with it. Some participants were using *Stay Strong* as a practitioner-led assessment and case-planning tool while others were encouraging clients, and in some cases family members, to use the app independently of the worker.

#### Online Health Prevention Programs

Participants reported using two culturally specific online health prevention programs—one aimed at smoking cessation and the other aimed at managing cannabis use.

#### YouTube

Participants used YouTube clips for health information, particularly information about alcohol and other drugs, and for music for relaxation purposes.

#### Self-Help Mobile Apps

Participants in this study used self-help relaxation and breathing apps for the management of stress.

Within the context of their practice and their interactions with clients, only eMH resources that could be accessed via a mobile device (either a smartphone or a tablet) were used. Most of the client interactions occurred in community settings and often occurred outside in parks or outside the building of the service where they worked. Some participants referred clients to eMH resources that require a desktop computer (for example *Mindspot*) and one participant used a desktop computer to access *Mindspot* for her own self-care. Table 1 reports on the types of eMH resources that were used for particular purposes.

**Table 1:** Types of eMental Health resources used.

Use	Type of eMH^a^
Inform and educate	Videos (for example, alcohol and other drug videos on YouTube), Facebook, mental health website (*beyondblue*), online treatment program (*Mindspot*), online health prevention programs (for example, to quit smoking, manage cannabis use)
Assess	*AIMhi Stay Strong* app
Case plan or manage	*AIMhi Stay Strong* app
Respond to crises	Online emergency telephone numbers and services
Refer to eMH resources	Mental health website (*beyondblue*), information websites, online services and contact information (for example, health and welfare services, grief, loss, suicide), mobile apps (for example, meditation, breathing)
Support social inclusion	Information websites, health and welfare services, music, videos
Care for self and family	Mobile apps (for example, meditation, *AIMhi Stay Strong*), online treatment program (*Mindspot*)

^a^eMH: eMental Health.

## Discussion

### Principal Findings

The findings from this exploratory study provide a detailed description of how one group of Aboriginal and Torres Strait Islander service providers report on how they have integrated eMH resources into their practice after an eMH training course, and the types of eMH resources that they are choosing to use with their clients.

In summary, the study found that 9 of the 16 participants had implemented eMH resources into their routine practice following an eMH training program. The findings demonstrate that participants were using eMH resources for informing and education, assessment, case planning and management, referral, responding to crises, supporting social inclusion, and self and family care. Participants chose a variety of types of eMH resources to use with their clients, both culturally specific and mainstream. While they referred clients to online treatment programs, they used only eMH resources designed for mobile devices in their face-to-face contact with clients.

The findings highlight the particular cultural and professional context within which Aboriginal and Torres Strait Islander service providers engage with their clients.

### Purpose of eMental Health Practice

In the absence of other studies of utilization of eMH by other health or related workforces, it is difficult to compare or contrast the findings from this study with other studies.

The only point of comparison that can be made is with a conceptual paper by Reynolds et al [31] that describes a set of clinical practice models to guide primary health care professionals and peer workers in the use of online assessment or screening and psychological treatment programs. The 5 clinical practice models are described on a continuum of therapist involvement: promotion, case management, coaching, symptom-focused treatment, and comprehensive therapy. Clinical practice models have become a popular benchmark for the development of clinical guidelines for the implementation of eMH across primary health care professions [32].

The participants in this study did report engaging in promotion and referral, case management and coaching with their clients, but symptom-focused treatment and comprehensive therapy lay outside both their expertise and their roles.

While they did refer clients to one online assessment or screening and psychological treatment program (*Mindspot*), participants in this study did not integrate this type of eMH resource into their practice.

Comparing the findings from this study with the conceptual clinical practice models described in Reynolds et al [31] is of limited use for 3 reasons. First, the primary target audience for the clinical practice models is allied health professionals working in clinical settings. While the Reynolds et al paper also includes peer workers and nonclinical workers, it assumes those workers are working under clinical supervision. Second, the models described in Reynolds et al [31] focus on the use of only one type of eMH resource—online assessment or screening and psychological treatment programs. Third, caution must be taken in assuming a commonality of practice across different professional contexts. Terms like coaching, for example, have different tacit meanings within different professional contexts. What coaching means to a clinical psychologist may be quite different to what coaching means to a community-based welfare worker.

### Types of eMental Health Resources

#### Overview

Overall, the findings of this study demonstrate a clear preference among this cohort for a variety of eMH resources that have been designed for or can be easily accessed through mobile devices (smartphones and tablets). This finding is in accord with findings from a number of studies that confirm the mobile smartphone with a prepaid service plan as the digital device of choice amongst Aboriginal and Torres Strait Islanders [33,34]. This preference is significant to our understanding of the choices that Aboriginal and Torres Strait Islander service providers and clients make about eMH resources, as it leads to eMH resources designed for mobile devices, resources that do not require long periods of time on the Internet, and resources that are not expensive to download.

#### Low-Intensity eMental Health Resources

Participants did not choose eMH resources designed to treat low-intensity mental health disorders like anxiety or depression. While it is not clear why this cohort did not use mainstream online symptom-based psychological programs, some inferences can be drawn from related evidence. One is that the service providers under study are not trained mental health specialists and, while their clients do present with mental health disorders, the treatment of those disorders falls outside their scope of practice [28]. Another is the reported discomfort Aboriginal people feel with the negative connotations that symptom-based mental health diagnoses carry as opposed to more culturally sympathetic conceptions of social and emotional wellbeing [7,25].

The only online treatment program to which workers referred clients was the *Mindspot* program, which offers the only culturally adapted version of a mainstream online assessment and treatment program (the *Mindspot Clinic Indigenous Wellbeing Course*). A recent study of the profile of users accessing the *Mindspot* website reports that Aboriginal and Torres Strait Islanders visited the site at a rate that closely matched national population statistics [35].

Some participants did use *Stay Strong* for client assessment and as a case management and care planning tool, as identified in Puzka et al [25]. This preference adds weight to the suggestion that eMH resources designed within a social determinants of social and emotional wellbeing framework may better capture Aboriginal conceptions of mental health than eMH resources based on biomedical models [25].

It is possible that if and when more culturally relevant treatment programs become available Aboriginal and Torres Strait Islander service providers may be more likely to refer their clients to these services or integrate them more fully into their case management models if they were to be given the training to do so.

The choice of mobile apps by participants in this study related only to the management of stress via relaxation and meditation apps. This preference may be explained by the high rates of psychological distress reported in the Aboriginal and Torres Strait Islander community and the high prevalence of stressors and stressful events in their lives [36]. It is also possible that these workers consider stress management to fall within their professional expertise and scope of practice, where the clinical management of anxiety and depression and other mental health disorders does not. There was no evidence that this cohort was using mobile apps for clinical purposes such as symptom assessment or the tracking of treatment progress.

#### Health Education and Health Promotion Resources

Bennett-Levy et al [28] highlighted the importance of matching types of eMH resources to particular work roles. The authors of this study queried the suitability of therapy-oriented resources for Aboriginal and Torres Strait Islander service providers, who may better be described as health educators than clinicians and therefore may be more comfortable using eMH resources with a health education or health promotion focus.

Participants in this study used a variety of types of eMH resources to inform and educate their clients about mental health and social and emotional wellbeing that included information websites, online health prevention programs, and YouTube videos. They also reported using Facebook as a means of keeping themselves updated and informed professionally.

Consistent with the findings from an earlier study [28], participants in this study had continued to implement Youtube clips into their practice. Rice et al [37] report a cultural compatibility between multimedia and the orally and visually oriented culture of Aboriginal and Torres Strait Islanders. Another study [38] reported on Aboriginal and Torres Strait Islander preferences for narrative story-telling approaches in Internet resources provided by government. The evidence suggests the potential that YouTube videos have for engaging Aboriginal and Torres Strait Islanders in eMH.

However, establishing an evidence base for the use of health information and health promotion resources will require researchers to broaden their focus from online treatment programs [28]. One systematic literature search found, for example, that of 60 indigenous health promotion tools available, only 11 had been evaluated for impact after implementation and only 5 of those reported strong impacts [39].

Overall, the findings from this study confirm a number of key features that may distinguish Aboriginal and Torres Strait Islander service providers from other health professionals. The contexts within which they work, the manner in which they use eMH resources, and the types of eMH resources they use with their Aboriginal and Torres Strait Islander clients all deserve separate and distinct investigation and reporting. To conflate these workers within a homogenized discourse about the implementation of eMH among health professionals is to miss the distinctive role they play and the unique opportunities they offer in the overall delivery of services to Aboriginal and Torres Strait Islander people.

### Limitations

This study has drawn on experiences and insights from Aboriginal and Torres Strait Islander service providers from one regional area of Australia. Given the diversity of roles and contexts of Aboriginal service providers across Australia [20], it is unclear to what extent the findings from this study can be generalized to urban or remote community contexts.

The participants in this study had all attended an eMH training workshop, and 13 of these participants had attended follow-up eMH consultation sessions. In the course of both the training workshop and the follow-up consultation sessions, participants were introduced to particular eMH resources. While participants did report independently searching for, choosing and implementing eMH resources, their training experience will have influenced the choice of eMH resources that they subsequently used in their practice 6 to 8 months later.

### Future Research

The findings from this small exploratory study need testing in different locations and with a national sample of Aboriginal and Torres Strait Islander service providers.

There is scant research that focuses on populations of consumers and their preferences for eMH services and resources [24]. One systematic review of studies of eMH services for anxiety and depressive disorders found that the current profile of users of these services is female, more educated, and socially advantaged [24]. In these studies ethnicity was infrequently reported. There is only one small study to date of Aboriginal and Torres Strait Islander acceptability of two culturally specific mental health apps [40], and one study of access and engagement rates by Aboriginal and Torres Strait Islander people of one online psychological assessment and treatment program [35]. More research needs to be conducted on the preferences of Aboriginal and Torres Strait Islander people for various types of eMH resources.

Opportunities may lie, for example, with the relative youthfulness of the Aboriginal and Torres Strait Islander population [3]. Young Aboriginal and Torres Strait Islanders are competent, confident, and frequent users of digital technology and social media [37]. They employ digital technologies and social media for culturally specific purposes including maintaining community and family and kinship connections and affirming cultural identity. In addition, a number of studies comment on the unrealized potential for health education and health promotion programs to harness this dynamic digital space [37,33].

### Conclusions

This paper demonstrates that the manner in which a particular workforce integrates eMH resources into their practice is highly contextual and suggests that a one-size-fits-all approach to eMH implementation is of limited value to the field. Workforces, and the professions within them, each have characteristics that will offer both opportunities and constraints to the manner in which they use eMH resources and the types of eMH resources they choose to use. At time of writing it seems particularly important that the eMH field expand its focus from allied primary health care professionals who work in clinical settings to workforces that are community-based and who may work outside the traditional health system. In particular, policy makers and workforce trainers need to recognize the diversity of workforces that support the social and emotional wellbeing of Aboriginal and Torres Strait Islander people and other minority populations.

The findings reported in this paper may be useful to those working within indigenous communities in postcolonial countries other than Australia. While eMH does not appear to have yet gained traction among indigenous communities in New Zealand or North America, eMH offers the global indigenous community an opportunity to share experiences and eMH resources across national borders.

This paper also highlights the importance of identifying and addressing the particular needs and user preferences for eMH of minority groups generally and in this case Aboriginal and Torres Strait Islander people. If eMH is to improve accessibility to and equity in the mental health system rather than further entrench existing inequalities, the field needs to foster the production of culturally relevant resources, services, and treatment modalities that both support social and emotional wellbeing and treat mental health disorders.

## Abbreviations

eMH: eMental Health
